# Short time administration of antirheumatic drugs - Methotrexate as a strong inhibitor of osteoblast's proliferation in vitro

**DOI:** 10.1186/1746-160X-8-26

**Published:** 2012-09-29

**Authors:** Tobias Annussek, Johannes Kleinheinz, Szuwart Thomas, Ulrich Joos, Kai Wermker

**Affiliations:** 1 Department of Cranio-Maxillofacial Surgery, University Hospital Muenster, Muenster, Germany; 2 Department of Cranio-Maxillofacial Surgery, Fachklinik Hornheide at the University of Muenster, Waldeyerstr 30, 48149, Muenster, Germany

**Keywords:** Antirheumatic drugs, Methotrexate, Osteoblast, In vitro, Bone metabolism

## Abstract

**Introduction:**

Due to increasing use of disease modifying antirheumatic drugs (DMARDs) as first line therapy in rheumatic diseases, dental and maxillofacial practitioner should be aware of drug related adverse events. Especially effects on bone-metabolism and its cells are discussed controversially. Therefore we investigate the in vitro effect of short time administration of low dose methotrexate (MTX) on osteoblasts as essential part of bone remodelling cells.

**Methods:**

Primary bovine osteoblasts (OBs) were incubated with various concentrations of MTX, related to tissue concentrations, over a period of fourteen days by using a previously established standard protocol. The effect on cell proliferation as well as mitochondrial activity was assessed by using 3-(4, 5-dimethylthiazol-2-yl) 2, 5-diphenyltetrazolium bromide (MTT) assay, imaging and counting of living cells. Additionally, immunostaining of extracellular matrix proteins was used to survey osteogenic differentiation.

**Results:**

All methods indicate a strong inhibition of osteoblast`s proliferation by short time administration of low dose MTX within therapeutically relevant concentrations of 1 to 1000nM, without affecting cell differentiation of middle-stage differentiated OBs in general. More over a significant decrease of cell numbers and mitochondrial activity was found at these MTX concentrations. The most sensitive method seems to be the MTT-assay. MTX-concentration of 0,01nM and concentrations below had no inhibitory effects anymore.

**Conclusion:**

Even low dose methotrexate acts as a potent inhibitor of osteoblast’s proliferation and mitochondrial metabolism in vitro, without affecting main differentiation of pre-differentiated osteoblasts. These results suggest possible negative effects of DMARDs concerning bone healing and for example osseointegration of dental implants. Especially the specifics of the jaw bone with its high vascularisation and physiological high tissue metabolism, suggests possible negative effects of DMARD therapy concerning oral and cranio-maxillofacial bone surgery as could be seen in a similar way in bisphosphonate related osteonecrosis of the jaw.

## Introduction

Rheumatoid arthritis (RA) is a chronic autoimmune disease, clinically characterized by chronic synovitis, serological abnormalities, acute-phase reactants, and symptoms like pain or stiffness, leading to a score ≥ 6 of 10 as established by the 2010 classification criteria of the American college of rheumatology and European league against rheumatism [1]. The prevalence in developed countries rages between 0,5 – 1,1% with an incidence of 0,02 to 0,07 per 1000 [2]. In spite of newer molecular and cellular understanding of RA the pathophysiological pathways and etiology of disease is not already understood in detail [3]. Impact of RA seems to be highly associated with genetic susceptibility, environmental factors and changes in mesenchymal tissue. As genetic factors association with human leukocyte antigen-DRB1 allels, the so called shared epitope could be verified. Those patients were positive for autoantibodies, the IgM and IgG rheumatoid factors, as well as antibodies against citrullinated peptids (ACPA), identified as directly acting against the Fc fragment of human IgG [4]. Most dominant environmental factors are smoking, age and gender (male / female ratio 1/3) [5]. Local Tissue consists of four major types of cells involved in rheumatoid synovial inflammation, the fibroblast-like cells, macrophage like cells as well lymphocytes (T and B Cells). Most insights indicate that RA starts in the joints with enhanced cytokine production by macrophage- and fibroblast-like synoviocytes. These cytokines activate pathways of the adaptive immune system especially targeting T-cell subsets and regulatory T-cells, leading to macrophages, chondrocytes and osteoclasts driven tissue damage [6]. However, the increasing age of patients is highly associated with the contract of RA and edentulism. Moreover, the growing evidence suggests an association between periodontal disease and systemic diseases such as rheumatoid arthritis [7]. Therefore, patients suffering from RA, show characteristics that confirm to lots of patients daily locating our hospital. Since the paradigm shift in RA therapy no longer the reduction of symptoms by the use of analgetics or anti-inflammatory drugs (NSAIDs) is the overarching principle. Actually treatment of disease is dominated by an aggressive and early use of disease modifying anti-rheumatic drugs as recommended by the ACR and EULAR [8]. Therefore, the availability of new therapies in rheumatoid arthritis, especially the admission of newer antirheumatic drugs increases remarkably during the last decades [9]. DMARDs are a heterogeneous group of agents, whose diverse mechanisms of action are not already understood. The most often and usually first time administered DMARD is methotrexate. MTX was primary used at high dosages (100-1000mg) in oncology as anti-neoplastic agent. First data of low dose MTX use in RA Therapy go back to the 1960s. In the following years doses ranged from 2,5-25 mg once weekly, administered orally, intravenously or even subcutaneously [10]. Actually it is still the anchor drug in RA Therapy, with mean dosage of 7,5 mg once weekly, whereas latest reports of the ACR and EULAR recommend higher dosages. However, the precise understanding of antirheumatic action in spite of antineoplastic action remains uncertain. It is known that high concentrations of MTX as a folic acid antagonist, inhibits the de novo purine and pyrimidine synthesis. Intracellular, a part of MTX undergoes polyglutamination (MTXglu). Both, MTX and MTXglu inhibit the dihydrofolate reductase (DHFR), thymidylat synthase and 5-aminoimidazole-4-carboxamide ribonucleotide (AICAR) transformylase, which leads to accumulation of extracellular adenosine by devious inhibition of AMP deaminase and primary derived from adenine nucleotides. This extracellular adenosine accumulation has been found out to mediate the anti-inflammatory effect of MTX [11-13]. Adverse reactions of MTX are well documented in literature, even if it is often described as generally well tolerated and to possess a superior safety profile [14,15]. In fact, side effects of DMARDs are discussed controversially. It is already recognized that high dose MTX therapy causes osteoporosis in predisposed patients [16]. Just rare data exist concerning metabolic changes impairing osteogenic pathways affected by low dose MTX therapy. However, one of the most important tissues, which is routinely affected by maxillofacial surgery in the context of fractures, orthognathic surgery, implantology or temporo-mandibular joint diseases is the jaw bone and therefore bone metabolism with its bone forming osteoblasts. This emphasizes the demand of a detailed knowledge of disease specific metabolic changes and pathogenesis, as well as potential side effects and adverse events of disease modifying antirheumatic drugs affecting cranio-maxillofacial surgery. Especially neurological, phylogenetic and metabolic specifics of the cranio-maxillofacial region amplify this request.

Therefore we investigate the proliferation, mitochondrial metabolism and differentiation of primary bovine osteoblasts on clinically relevant concentrations of MTX, to determine potential side effects and risks of low dose methotrexate on the osseous structures of the oral cavity and facial region.

## Materials and methods

### Cell culture

Osteoblasts were cultured from the periosteal layer of calf metacarpals according to the method of Jones et al. [17]. In detail, 4x3mm pieces of periosteal layer where seeded into culture dishes of 136 mm diameter (TPP AG, Trasadingen, Switzerland) with the osteogenic layer facing downwards to afford the osteoprogenitor cells to migrate from the tissue explants. After one week, the pieces were removed and primary cells were cultured for three more weeks by using High Growth Enhancement Medium (MP Biomedicals, Eschwege, Germany), supplemented with 10% foetal calf serum (Biochrom KG, Berlin, Germany), 10.000 IU/ml penicillin, 10.000 μg/ml streptomycin and 250 μg/ml amphotericin (Biochrom KG, Berlin, Germany) at 37°C and 5% C0_2_ in humidified air. When primary osteoblasts reached confluence they were washed three times with phosphate-buffered-saline and harvested by ten minutes of incubation with accutase (PAA Laboratories, Cölbe, Germany). Afterwards, cells were pelleted by centrifugation. Cell number and size were determined using an electric field multi-channel cell counting system (CASY I, Schärfe System, Reutlingen, Germany). The resuspended cells were seeded into 24 well-plates (TPP AG, Trasadingen, Switzerland) at concentrations of 1x10^4^ cells per well and incubated for two more weeks with a solution of culture medium supplemented with MTX concentrations from 1 to 1000nM, according to MTX concentrations found in cortical bone of RA patients [18]. Additionally cells were cultured under same conditions on culture dishes of 87.2 mm diameter (NUNC, Langenselbold, Germany). Control samples, cultured in medium without MTX, were done for each point of investigation. An exponential dilution series of cells with 1 × 10^6^ cells per well as a starting point, were used to check methodology and to ensure that cells used for experiments were in the exponential growth phase. Since the beginning of the experimental procedure cell morphology was monitored daily by phase-contrast light microscopy. All samples were done independently in double triplicates (total n = 516). Medium was changed twice weekly.

### Cell Counting

After day 1, 3, 6, 10 and 14 post MTX addition cell proliferation was measured by standardised taking digital photo (NIS Elements 2.20, Nikon Instruments Inc., Melville, NY, USA) of each well and counting of living cells per unit area using Java-based image processing program (Image J Cell Counter, National Institute of Health, USA). The procedure was performed by two different and blinded examiners (n = 300).

### MTT-assay

To determine cell viability and mitochondrial activity we performed a 3-(4,5-dimethylthiazol-2-yl) 2,5-diphenyl-tetrazolium bromide (MTT) assay (SigmaAldrich Co., St. Louis, MO, USA). At the same days, when digital photos were taken, cells were incubated for one hour with 1 ml MTT solution (0,25 mg/ml). After incubation-time, medium was aspirated and 200 μl of propanol was added to lyse the cells and dissolve the released formazan crystals (n = 150). The extinction was measured at a wavelength of 570 nm using an enzyme-linked immunosorbent assay reader (μQuant, Biotek instruments, Bad Friedrichshall, Germany).

### Immunostaining

The additional culture dishes were used to characterize osteoblasts by immunostaining. When control samples reached confluence, the expression of collagen I, osteocalcin and osteonectin was determined. Therefore cells were washed two times with phosphate buffered saline and fixated for 20 minutes at −20°C. Blocking solution (CANDOR Bioscience, Wangen, Germany) was used for 15 minutes, before 60 minutes of incubation with primary antibodies (collagen I 488 rabbit, Biotrend, Köln, Germany; osteocalcin 488 mouse and osteonectin 488 mouse, TaKaRa, Saint-Germain-en-Laye, France). Secondary antibodies (Biochrom K4000, 4002, Biochrom KG, Berlin, Germany) were used according to the manufacture`s instruction (n = 30).

### Critical concentration identification

After first analysis of data of cell counting and MTT – assay, we saw an inhibitory effect of MTX on osteoblasts for all administered concentrations from 1 to 1000 nM compared to control without MTX, as described below in the results chapter in detail. Due to this fact, further experiments were performed to evaluate the critical value of that MTX-concentration, in which no more inhibition of proliferation or mitochondrial activity could be assessed. Osteoblasts were seeded into 24 well-plates (TPP AG, Trasadingen, Switzerland) at concentrations of 1x10^4^ cells per well as described before and incubated for one week with a solution of culture medium supplemented with MTX concentrations from 0,0001 to 1 nM (n = 36).

### Statistical analysis

The effect of MTX on proliferation and viability, respectively mitochondrial metabolism of osteoblasts were evaluated by using analysis of variance (ANOVA, post hoc Tamhane T2 – test) to distinguish between groups of different MTX-concentrations. Interrater reliability between the two examiners was tested calculating Cohen's kappa. All statistical analyses were performed using SPSS software (version 15.0; SPSS Inc., Chicago, Illinois, USA).

## Results

### Cell morphology and immunostaining

**Figure 1:**
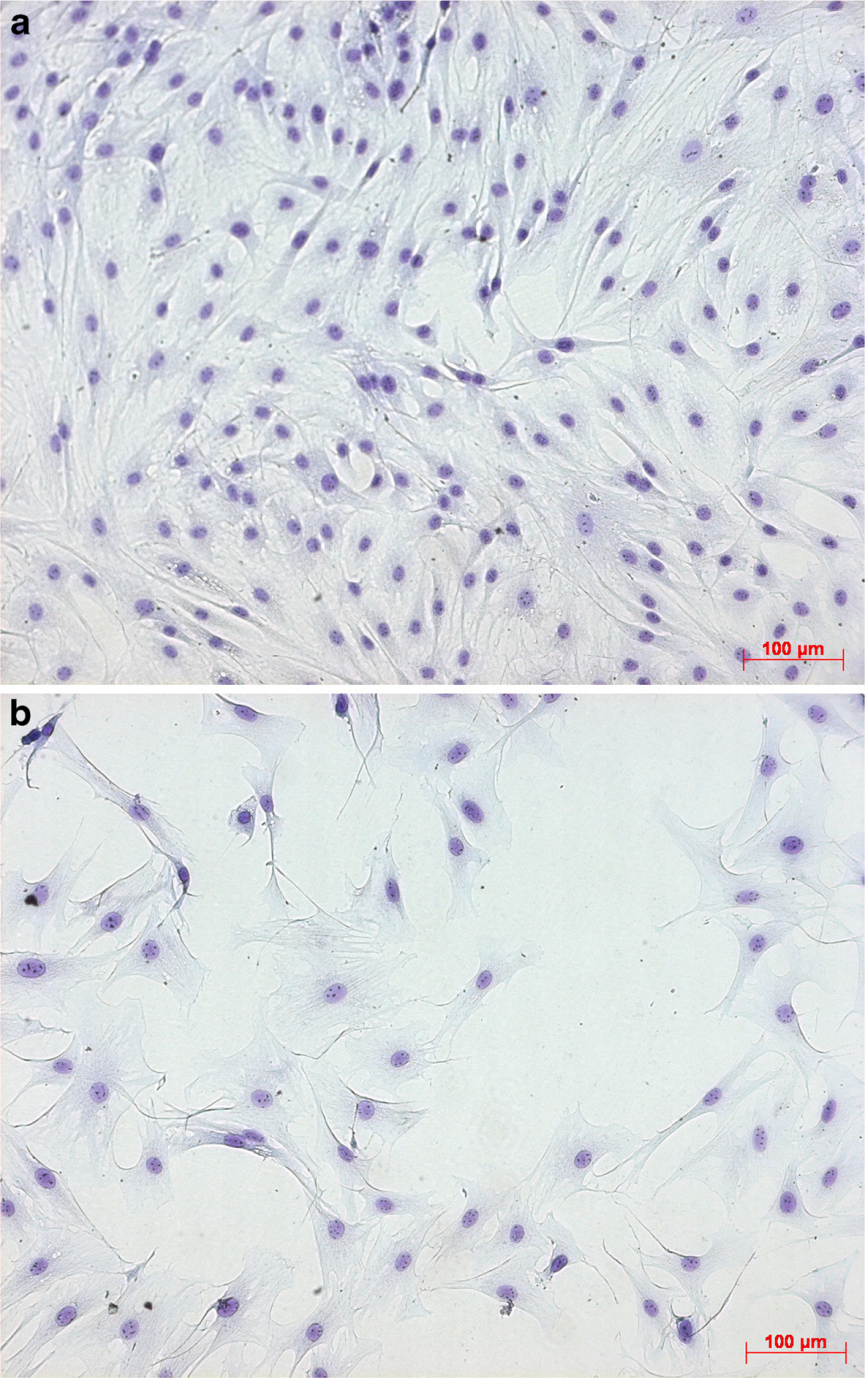
**a. Richardson – staining at Control without MTX (day 7, 10x).b**. Richardson – staining at 1000 nM MTX (day 7, 10x).

At the beginning of experimental procedure cell morphology was measured by daily phase contrast microscopy and adjudged as unaltered. The cultured cells showed typical characteristics of osteoblasts without any identifiable changings. Cells were displayed with flattened, spindle shaped bodies and bipolar processes. At starting point, mean diameter of OBs was 16,72 μm , assessed by electronic particle counter (CASY I TT, Schärfe System GmbH, Reutlingen, Germany). 72 h after incubation with MTX concentrations from 1-1000nM first changes could be observed in cultures, substituted with 100nM and 1000nM. Osteoblasts of the MTX group in contrast to the control group without MTX-addition, were less proliferating, as seen by the bright gaps between the cells, and developing longer cell-processes to get in contact with each other (Figure 1a, 1b). At day 6 these differences appeared at all MTX concentrations without noticeable discrepancy within MTX groups. Moreover, till day 14, cells which were incubated with different MTX concentrations, developed more voluminous cell bodies.

**Figure 2:**
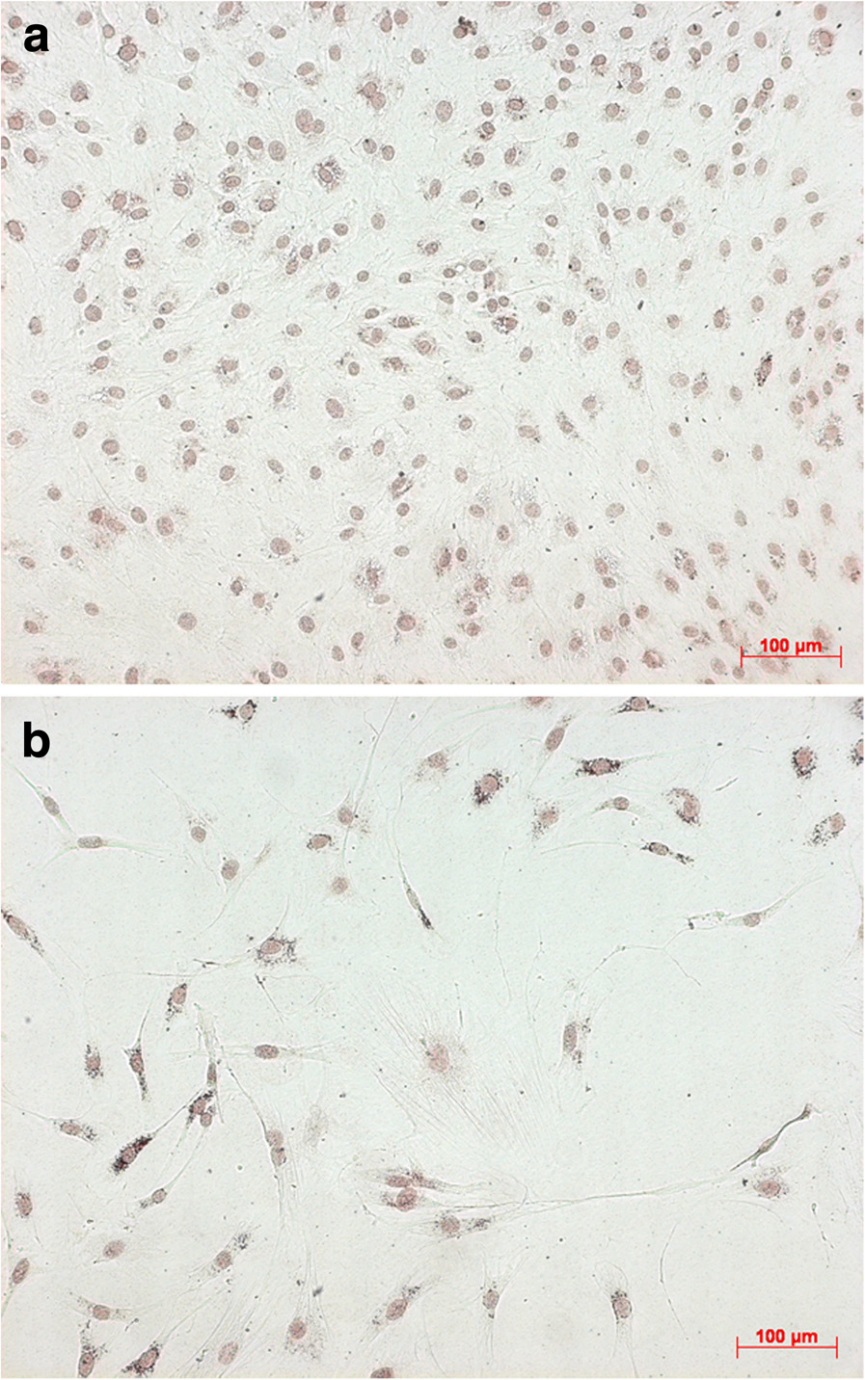
**a. Osteocalcin – immunostaining at Control without MTX (day 7, 10x).b**. Osteocalcin – immunostaining at 1000 nM MTX (day 7, 10x).

**Figure 3:**
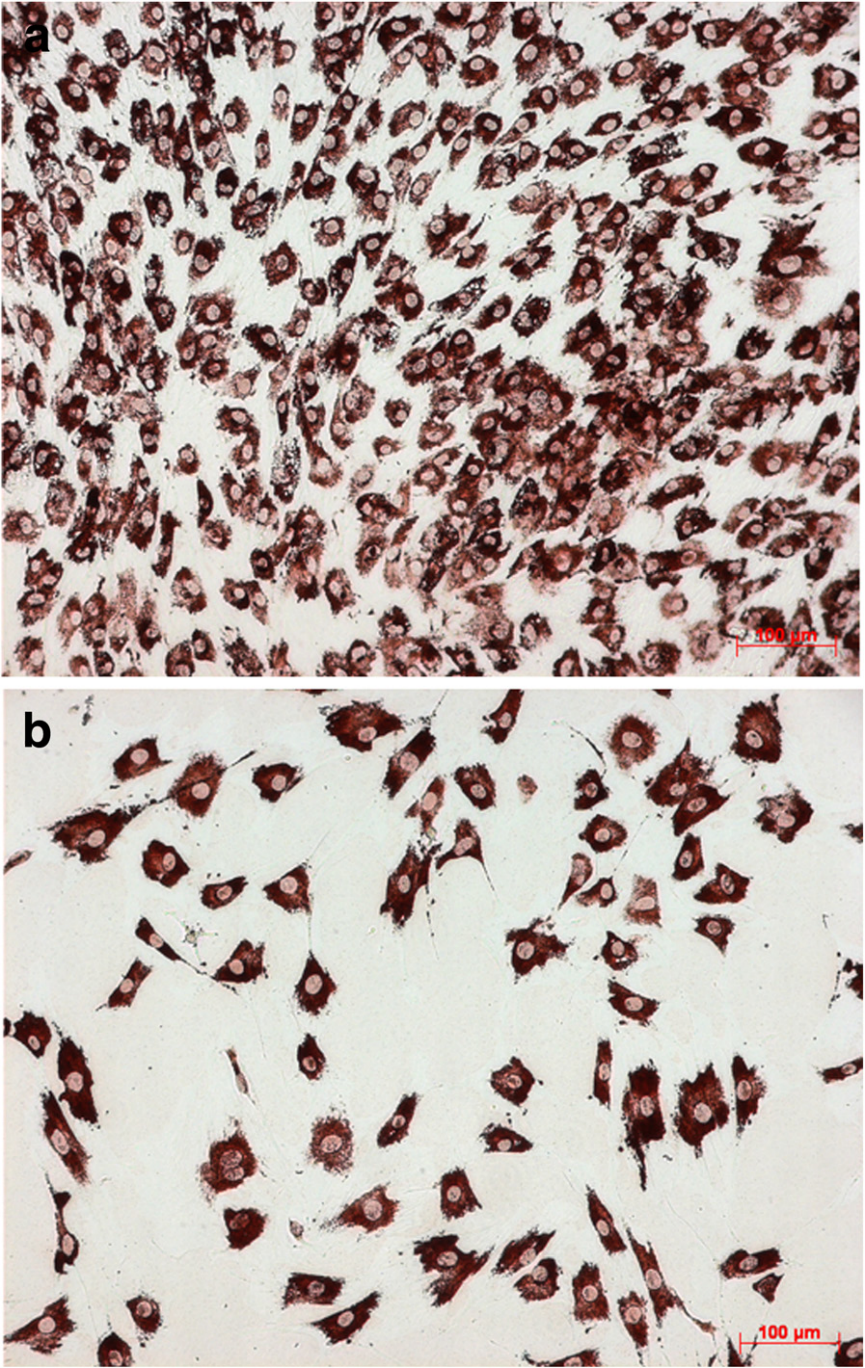
**a. Collagen I – immunostaining at Control without MTX (day 7, 10x).b**. Collagen I – immunostaining at 1000 nM MTX (day 7, 10x).

The expression of the osteogenic proteins, more precise collagen I, osteonectin, and osteocalcin, was not altered in general by addition of MTX (Figures 2a, 2b, 3a, 3b). At all samples bone specific proteins were detectable till the end of experimental procedure, even if the quantitatively observed expression seems to be decreased.

### Cell counting

**Figure 4:**
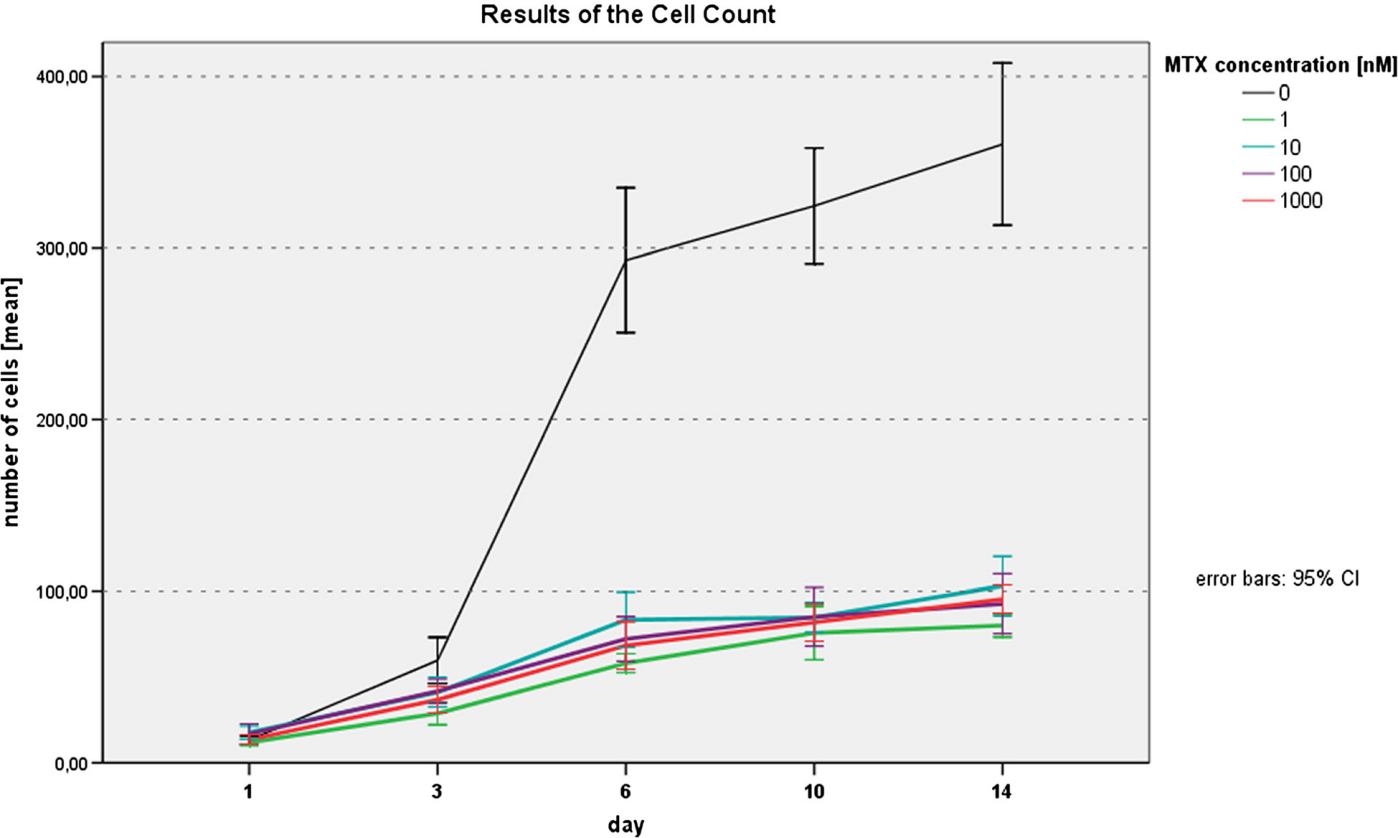
Results of Cell Counting.

After 24 h of incubation, number of cells attached on the polystyrene surfaces per unit area, were relatively constant between MTX and control groups. With p-values ≥ 0.085 no statistical significance was observed neither between MTX concentrations nor to control group. At day 3 we firstly found significant decrease in cell number concerning wells incubated with 1nM (p = 0.03) and 1000nM (p = 0.042) (Table 1). Moreover, no significant changes in cell number within the MTX concentrations from 1-1000nM could be observed. After six days of MTX incubation highly significant differences were found between all MTX concentrations compared to control (p ≤ 0.001). This inhibitory effect of MTX was seen also at day 10 at the same level of high significance and lasted until day 14, when differences to control group in cell numbers per unit reached a maximum (Figure 4). In addition, significant differences in cell numbers occurred between MTX concentrations of 1nM and 1000nM (p = 0.041). For all measurements, interexaminer reliability was high with Cohen's kappa κ = 0.937.

**Table 1:** Results of Cell Counting

			** MTX – concentration**			
		**Control**	**1 nM**	**10 nM**	**100 nM**	**1000 nM**
**Day 1**	mean	13.08	11.76	17.52	16.67	13.31
	SD	3.94	1.86	3.62	5.47	2.58
**Day 3**	mean	59.61	28.70	41.06	41.61	36.62
	SD	21.22	6.28	8.22	6.59	7.42
**Day 6**	mean	293.32	58.05	83.31	72.16	68.32
	SD	40.27	5.25	15.41	12.28	13.25
**Day 10**	mean	324.62	75.65	84.67	85.07	81.83
	SD	32.17	14.95	8.31	16.35	10.49
**Day 14**	mean	360.71	80.08	103.00	92.62	95.29
	SD	45.09	6.45	16.51	16.73	7.74

### MTT-assay

**Figure 5:**
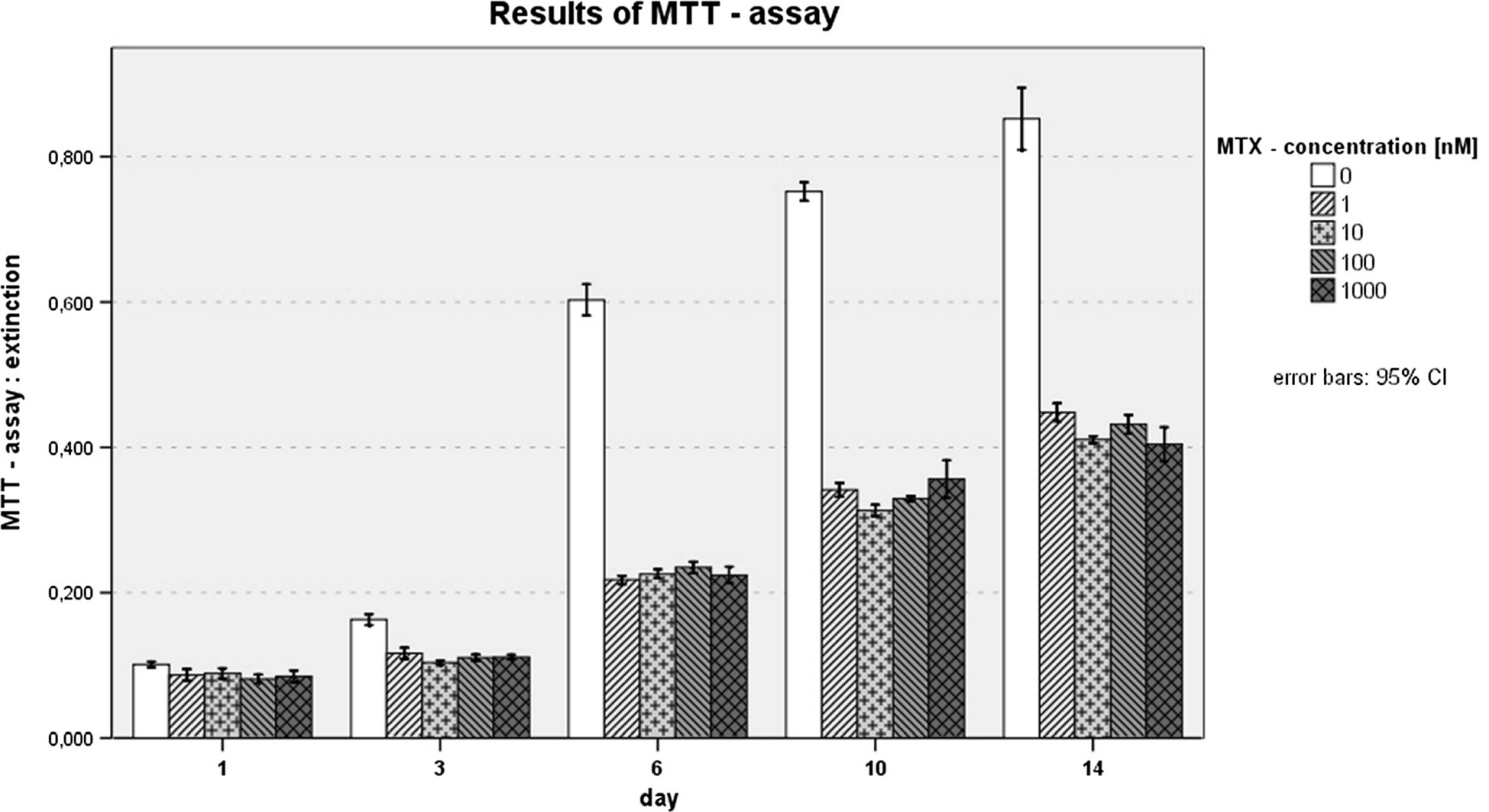
Results of the MTT – assay.

The first assay was performed 24h after methotrexate administration to ensure that osteoblasts were attached to polystyrene well surfaces as proven before. In spite of MTX groups, within no statistical significant differences in extinction values were found, all MTX concentrations showed significant lower extinction and therefore decrease of detectable mitochondrial activity compared to cells without MTX addition. Within MTX concentrations of 1nM - 10nM as well as 1000nM p-value was between 0.012 and 0.039. Concerning MTX concentration of 100nM, highly significant reduction of cell viability could be observed during the first day of incubation. From the beginning of day 3, extinction values started to differ within MTX groups but without straight proportionality to concentration. Reduction of mitochondrial activity was however highly significant to control (p ≤ 0.001). Till day 10 varying decrease of viability was measured and stayed at high level of significance. Then differences between extinction of the MTX groups themselves started to became significant (Table 2, Figure 5). Between 1nM/10nM, 10/100nM and 1-1000nM/0nM p-value was between 0.007 and ≤ 0.001. The maximum of reduction was found at day 14 between 10nM and control, with a mean difference of 0.439 of extinction. In comparison of cell number and mitochondrial activity (MTT-assay), the average extinction per cell decreases statistically significant within MTX groups.

**Table 2:** Results of measurement of MTT – assay extinction values

			** MTX – concentration**			
		**Control**	**1 nM**	**10 nM**	**100 nM**	**1000 nM**
**Day 1**	mean	0.101	0.087	0.089	0.081	0.085
	SD	0.009	0.013	0.011	0.009	0.013
**Day 3**	mean	0.163	0.117	0.104	0.110	0.111
	SD	0.018	0.012	0.005	0.007	0.005
**Day 6**	mean	0.603	0.218	0.226	0.234	0.224
	SD	0.051	0.009	0.010	0.012	0.018
**Day 10**	mean	0.752	0.341	0.313	0.329	0.356
	SD	0.031	0.015	0.012	0.005	0.040
**Day 14**	mean	0.852	0.448	0.411	0.432	0.404
	SD	0.102	0.019	0.008	0.021	0.037

### Detection of critical MTX-concentration

**Figure 6:**
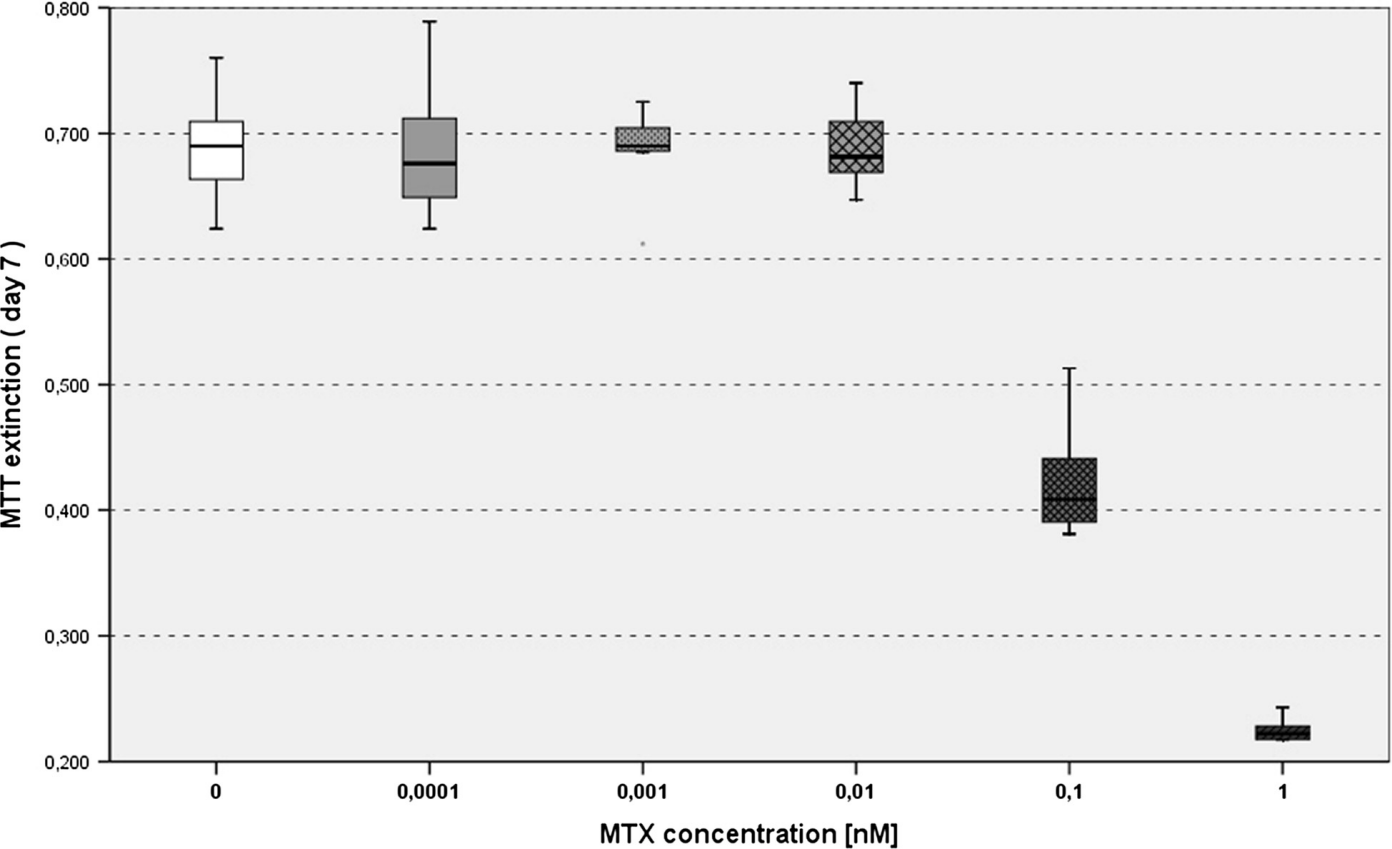
**Determination of critical inhibitory MTX – concentration.** Results of the MTT – assay at day 7.

MTX-concentrations of 1nM and 0.1nM showed still statistical significant inhibition of osteoblast`s proliferation and viability. Concentrations equal to or lower than 0.01nM MTX showed no inhibitory effects concerning cell proliferation, determined by cell counting, and mitochondrial metabolism, measured using the MTT-assay. From the beginning of day 7, differences between various MTX levels reached highest levels of statistical significance (Table 3). Between concentrations of 0.0001nM to 0.01nM no differences between MTX groups themselves neither to control group (no MTX, i.e. 0nM) could be found. Administration of 0.1nM MTX lead to highly significant reduction of measured MTT-absorption (p ≤ 0.001) compared to control and concentrations of 0.0001-0.01nM MTX. Mitochondrial activity, as measured by MTT-assay, showed further significant inhibition by MTX at a concentration of 1nM compared to 0.1nM MTX (p ≤ 0.001) (Figure 6).

**Table 3:** Results of MTT-assay at day 7 to determine the critical MTX-concentration

**MTX concentration [nM]**	**MTT extinction**		
	**mean**	**SD**	**95% Confidence interval**
**0 (Control)**	0.687	0.033	0.673 – 0.701
**0,0001**	0.684	0.046	0.655 – 0.713
**0,001**	0.690	0.028	0.673 – 0.708
**0,01**	0.689	0.029	0.671 – 0.707
**0,1**	0.424	0.044	0.396 – 0.453
**1**	0.213	0.031	0.193 – 0.233

## Discussion

The aim of this study was to investigate, whether low dose MTX causes changes in osteogenic differentiation, proliferation and metabolism. Other authors previously investigated the effect of low dose MTX on mouse osteogenic cell line (MCT3-E1), human osteoblastic osteosarcoma cells (UMR-106), human bone marrow stromal cells (BMSC) or human bone derived cells (HBDC) with controversial results [19,20]. Uehara et al. suggested that MTX inhibits the differentiation of early osteoblastic cells, without effecting proliferation of late-stage osteoblasts. In spite of this, Minaur and colleges observed that concentrations of ≥10nM MTX inhibits the proliferation of primitive marrow stromal cells but did not alter their maturation. Moreover, they found no influence of MTX on cells of the osteoblast linage, neither in differentiation nor in proliferation. In fact we firstly described a strong and dose independent reduction of middle-stage differentiated, bovine osteoblast`s cell proliferation by a variety of experimental methods at therapeutically relevant MTX concentrations of 1-1000nM found in cortical bone by Bologna et al. [18]. In our study, we could clearly demonstrate that even lower concentrations of MTX within common therapeutical margins led to significant decrease of osteoblast’s proliferation and metabolism. The apparent discrepancy to earlier studies could be explained by methodological diversity, implicating the MTT-assay being more sensitive compared to electronic particle counter. Moreover time of incubation seems to be an important variety. Even if we could observe differences between MTX concentrations of 1-1000nM, there was no straight proportionality, so that a dose independent effect is assumable within these ranges of MTX-concentration. Also a low concentration of 0.1nM MTX showed still significant inhibitory effects on osteoblast's, proliferation and mitochondrial metabolism. More over the incidence of interaction appeared rapidly. We determined a critical value of 0.01nM MTX and below in which no more impairments of osteoblast's, proliferation and viability could be detected. These concentrations are more than hundredfold lower than levels measured in RA patients receiving MTX in therapeutical dosages and more than a thousand times lower than critical inhibitory values of MTX concentration described before [21]. In our point of view, this is also a proof that the methods used in this study, especially the MTT-assay, feature even detection of effects on cells at very low pharmacological concentrations of administered agents. We agree with published data from Scheven et al., that osteogenic differentiation and synthesis of proteins of the primary cells are not affected, as shown by immunostaining. In fact primary cells are able to react sensitively to minor alterations of their surrounding [22]. This is why in our opinion the use of primary cells as drug testing system seems to be the most advisable to detect potent adverse reactions concerning bone in general and also of the bone in the cranio-maxillofacial region in particular. Certainly, bone metabolism is also dependent on osteoclasts, but evidenced based data are already missing to assess their behaviour while MTX incubation.

We pointed out the possible inhibitory effect of MTX on osteoblast`s proliferation and metabolism. This could be an important finding concerning mechanism affecting bone development, bone regeneration and bone healing. Of special interest in the oral and maxillofacial field are possible negative effects of MTX on bone healing after tooth extractions, bony resections, and augmentations in reconstructive surgery. However, evidenced based recommendations for perioperative use of MTX are barley available [23]. Concerning oral rehabilitation of RA patients with the help of dental implants, it has to be considered that reduced proliferation of bone cells like osteoblasts maybe associated with reduced osseointegration of dental implants. Controversially discussed clinical questions, e.g. if MTX means a contraindication for dental implantations or if special protocols with regard to prolonged osseointegration time have to be developed, have to be left unanswered because of lacking experimental and clinical evidence. Nevertheless, more in vitro and in vivo as well as clinical data have to be collected to give a precise appraisal of surgical outcome or failure concerning the oral and maxillofacial region.

## Abbreviations

ACPA: Antibodies against citrullinated peptids; ACR: American college of rheumatology; AICAR: 5-aminoimidazole-4-carboxamide ribonucleotide; AMP: Adenosine 5`-phosphate; BMSC: Bone marrow stromal cells; DHFR: Dihydrofolate reductase; DMARD: Disease-modifying-anti rheumatic-drugs; EULAR: European league against rheumatism; HBDC: Human bone derived cells; MTT: 3-(4, 5-dimethylthiazol-2-yl) 2, 5-diphenyltetrazolium bromide; MTX: Methotrexate; NSAIDS: Non-steroidal anti-inflammatory drugs; OB: Osteoblast; RA: Rheumatoid arthritis.

## Competing interests

The authors declare that they have no competing interests.

## Authors’ contributions

TA established experimental procedures, did acquisition and interpretation of data and drafted the manuscript JK helped to design and to coordinate the study ST gave technical assistance and helped to draft the manuscript UJ has given final approval of the version to be published KW has made substantial contributions to conception and design of the study and preformed the analysis of the data All authors read and approved the final manuscript.
